# Spread of the FAR-MRSA clone, a fusidic acid- and meticillin-resistant *Staphylococcus aureus* ST121, Europe, 2014 to 2024

**DOI:** 10.2807/1560-7917.ES.2025.30.28.2500452

**Published:** 2025-07-17

**Authors:** Louise Roer, Nicolas Yin, Olivier Denis, Karuna EW Vendrik, Romy D Zwittink, Daan W Notermans, Monique Perrin, Kirstin Khonyongwa, Anne Tristan, Benjamin Youenou, Franziska Layer-Nicolaou, Guido Werner, Hege Enger, Emily Charlotte Henry Eikrem, Jessica Darenberg, Barbro Mäkitalo, Magnus Paulsson, Jonas Björkman, Hong Fang, Erika Tång Hallbäck, Martin Sundqvist, Laura Lindholm, Kartyk Moganeradj, Silvia García-Cobos, Javier E Cañada-García, Barbara Juliane Holzknecht, Helle Brander Eriksen, Morten Hoppe, Mette Damkjær Bartels, Jose Alfredo Samaniego Castruita, Tinna Ravnholt Urth, Anders Rhod Larsen, Andreas Petersen

**Affiliations:** 1 Statens Serum Institut, Copenhagen, Denmark; 2 National reference centre for Staphylococcus aureus and other species, Department of microbiology, Laboratoire Hospitalier Universitaire de Bruxelles – Universitair Laboratorium Brussel (LHUB-ULB), Université libre de Bruxelles, Brussels, Belgium; 3 National Institute for Public Health and the Environment (RIVM), Bilthoven, the Netherlands; 4 Laboratoire National de Sante, Dudelange, Luxembourg; 5 Centre National de Référence des Staphylocoques, Hospices Civils de Lyon, Lyon, France; 6 Division Nosocomial Pathogens and Antibiotic Resistances, Department of Infectious Diseases, National Reference Centre for Staphylococci and Enterococci, Robert Koch Institute, Wernigerode Branch, Wernigerode, Germany; 7 The Norwegian MRSA Reference Laboratory, Department of Medical Microbiology, St. Olavs Hospital, Trondheim University Hospital, Trondheim, Norway; 8 The Public Health Agency of Sweden, Solna, Sweden; 9 Medical faculty, Lund University, Lund, Sweden; 10 Region Skåne, Lund, Sweden; 11 Karolinska University Hospital, Stockholm, Sweden; 12 Department of Infectious Diseases, Institute of Biomedicine, Sahlgrenska Academy, University of Gothenburg, Gothenburg, Sweden; 13 Department of Laboratory Medicine, Clinical Microbiology, Faculty of Medicine and Health, Örebro University, Örebro, Sweden; 14 Finnish Institute for Health and Welfare, Helsinki, Finland; 15 UK Health Security Agency, Colindale, England; 16 Laboratorio de Referencia e Investigación en Resistencia a Antibióticos e Infecciones Relacionadas con la Asistencia Sanitaria, Centro Nacional de Microbiología, Instituto de Salud Carlos III, Madrid, Spain; 17 Copenhagen University Hospital—Herlev and Gentofte, Herlev, Denmark; 18 Department of Clinical Medicine, University of Copenhagen, Copenhagen, Denmark; 19 Copenhagen University Hospital – Amager and Hvidovre, Hvidovre, Denmark; * These authors contributed equally to this work and share last authorship.

**Keywords:** Methicillin-resistance, fusidic acid resistance, Staphylococcus aureus, impetigo, multi-resistance, international clone

## Abstract

We describe the genetic characteristics of a fusidic acid- and meticillin-resistant *Staphylococcus aureus* (MRSA) clone widespread in Europe, based on whole genome sequences from 317 isolates. The clone is causing impetigo and other skin and soft tissue infections, primarily in young children. Comparison with publicly available *S. aureus* ST121 sequences showed that the clone is clearly distinct from previously described ST121 clones. European and other international readers should be aware of the emergence of this community-acquired MRSA clone.

Meticillin-resistant *Staphylococcus aureus* (MRSA) is notifiable in Denmark, where all new isolates, including infections and screening samples, are sent to the national reference laboratory for typing. In June 2023, a fusidic acid-resistant (FAR) MRSA clone of sequence type 121 (ST121) was isolated for the first time in Denmark from impetigo cases in a kindergarten and family members. Further cases involving this clone were observed in summer 2024, again in children presenting with impetigo. Genetic markers of the outbreak isolates resembled those described in the European epidemic fusidic acid-resistant impetigo clone (EEFIC) of meticillin-sensitive *S. aureus* (MSSA). The EEFIC has been causing epidemics in Europe since the beginning of the millennium [1], and FAR-MRSA isolates also resembled recently described fusidic acid-resistant MRSA and MSSA impetigo isolates from the Netherlands and Belgium, respectively [2,3]. Our aim was to provide more details about the current situation and see how far the clone has spread in Europe.

## Characterisation of the clone

We posted a message on EpiPulse (2024-ARH-00016), and responding European national reference laboratories for MRSA surveillance were invited to share whole genome sequences and available clinical information from isolates with the same properties. We reconstructed a phylogeny based on single-nucleotide polymorphism (SNP) and screened the genomes for resistance genes and virulence markers.

**Figure 1:**
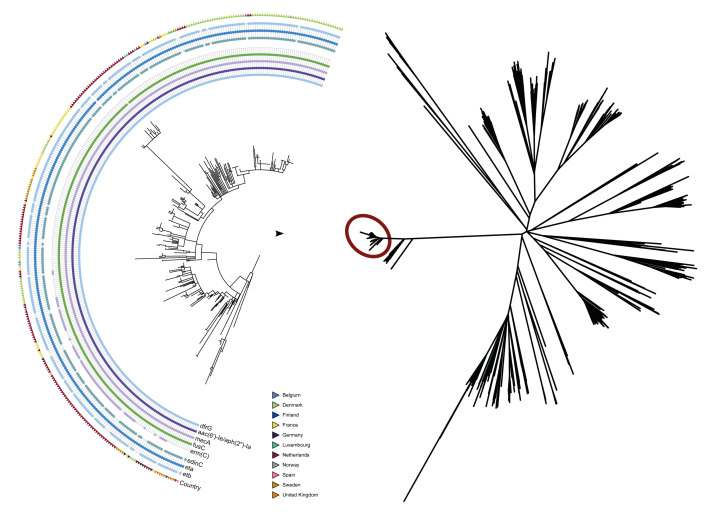
Single nucleotide polymorphism phylogeny of *Staphylococcus aureus* ST121, sequences from European reference laboratories and the NBCI database, 1932–2024 (n = 1,188)

The FAR-MRSA clonal group consisted of 317 isolates belonging to ST121/clonal complex (CC)121, with *spa* type t272 or closely related variants. In Supplementary Table S1, we append additional information on the isolates, country and date of isolation, sex and age group of cases, whether the sample was from an infection or screening, and who submitted the isolates. The clone in general shared previously described impetigo markers found in EEFIC (*eta*, *etb*, *edinC*), encoding exfoliative toxin A and B and epidermal cell differentiation inhibitor C, respectively (Figure 1). The majority of isolates carried *fusC*, *dfrG* and *aac*(6')-Ie/*aph*(2”)-Ia genes, encoding fusidic acid, trimethoprim and gentamicin resistance, respectively. All isolates were phenotypically resistant to fusidic acid. Sixty- four of the 133 Dutch isolates and six isolates from other countries (two each in France and Germany, one each in Belgium and Denmark) additionally harboured the *ermC* gene, encoding macrolide, lincosamide and streptomycin B resistance (Figure 1). The new FAR-MRSA clone had a different fusidic acid resistance gene, *fusC*, rather than the *fusB* present in the EEFIC and Belgian MSSA clones [1,2]. Both genes belong to the FusB family, which prevents fusidic acid from interacting with translation elongation factor G [4].

NCBI: National Center for Biotechnology Information.

Sequences from 11 European national reference laboratories for surveillance of methicillin-resistant *Staphylococcus aureus*, historical isolates of ST121 and downloaded *S. aureus* ST121 genomes from NCBI are included. On the right, all isolates are included, on the left, the 317 isolates that constitute the FAR-MRSA clone. In the circle in the left part, country of isolation, selected resistance genes and selected virulence genes are shown.

We downloaded 781 *S. aureus* ST121 whole genome sequences from the National Center for Biotechnology Information (NCBI) and compared them to the 407 collected sequences, including the 317 FAR-MRSA isolates. Detailed listings of the 317 isolates belonging to FAR-MRSA are appended in Supplementary Table S1. Supplementary Table S2 provides accession numbers and location of isolates for the 781 downloaded sequences, and Supplementary Table S3 contains information on collected *S. aureus* ST121 that were not part of the FAR-MRSA. The SNP phylogeny was based on 87,238 phylogenetically informative SNPs. The FAR-MRSA isolates formed a distinct clade comprising 317 isolates from 11 countries with 0–85 SNPs. A cluster of 26 *fusC*-negative MRSA isolates showed pairwise distances of 87–195 SNPs to the FAR-MRSA isolates and were thus not closely related. They originated from 10 countries in Europe, Africa, the Arabian Peninsula, the United States and Australia and were isolated between 2015 and 2024. Apart from that cluster, the most closely related isolate was 517 SNPs away from the FAR-MRSA cluster. The phylogeny included EEFIC strains (n = 7) and Belgian MSSA isolates (n = 30), all of which were genetically distant from the FAR-MRSA cluster, with a minimum of 662 and 626 SNPs, respectively. Among the 871 isolates not belonging to the clone, we found acquired fusidic acid resistance genes, *fusB* and *fusC*, in 101 isolates (12%). Zooming in on the FAR-MRSA, we saw geographical clustering but also found closely related isolates across multiple countries (Figure 1).

## Demographic and clinical characteristics

**Figure 2:**
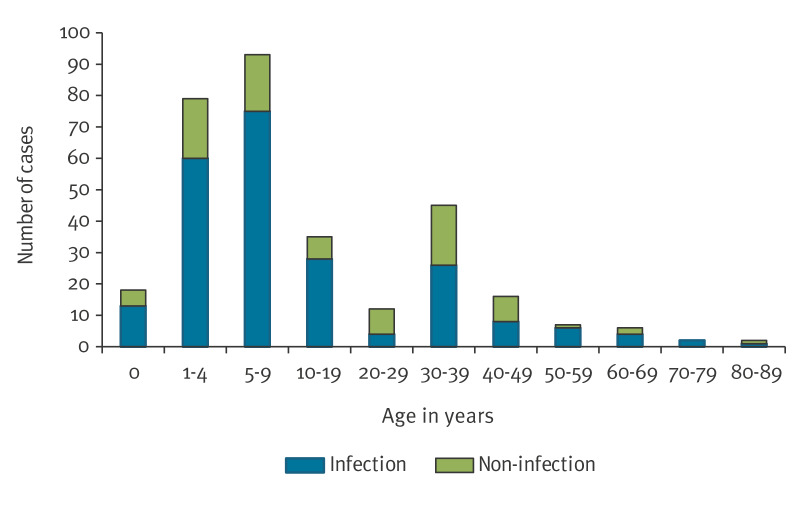
Age distribution of cases with FAR-MRSA, divided into infections and non-infections (screening samples), 11 European countries, 2014–2024 (n = 315)

Half of the isolates were from children aged 0–9 years (Figure 2) and 94% of those presented with an infection. The patients in the age group 30–39 years most probably represented parents of affected children who were positive at the screening of family members. However, almost two-thirds in this age group also had an infection at the time of sampling. In addition to impetigo, the FAR-MRSA was reported in other clinical skin and soft tissue infections such as exfoliative dermatitis and wounds.

Age information not available for two cases.

## Spread and origin

**Figure 3:**
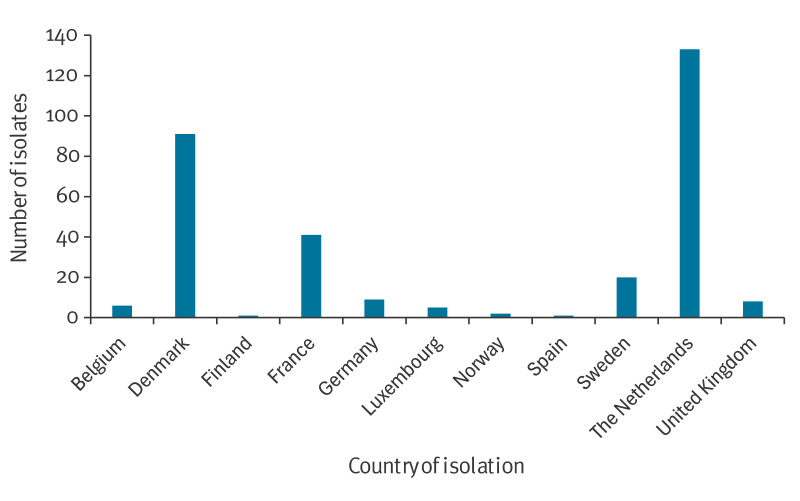
Number of isolates belonging to the FAR-MRSA clone, by country of isolation, 11 European countries, 2014–2024 (n = 317)

The described FAR-MRSA clone was reported from 11 European countries (Figure 3). Two additional countries reported that they did not have any isolates with the FAR-MRSA characteristics. The earliest two isolates in this investigation belonging to the clone were from 2014, one from the Netherlands and one from Germany. The number of SNPs between the 317 isolates suggest a close genetic relationship, but only little epidemiological information was available on cross-border transfer. For seven of the cases from the Netherlands travel abroad were reported, to destinations including Germany, Morocco and the Maldives [2]. One of the isolates from Luxembourg was from a patient reporting recent holiday travel to the Netherlands. At least 14 of the 20 Swedish patients had reported recent travel to Spain, Portugal or Qatar. The single isolate from Finland was obtained from a British tourist and it clustered closely together with the eight isolates from the United Kingdom (Figure 1). Most samples (202 of 275 where information was available) were submitted by general practitioners from non-hospitalised patients, suggesting community acquisition of the clone. 

## Seasonality

**Figure 4:**
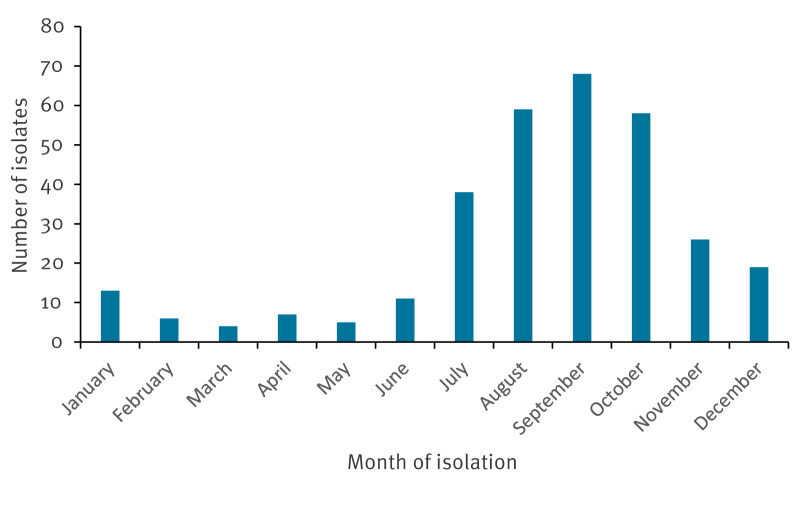
Number of isolates per month for all cases with available date of isolation, irrespective of year and country, 11 European countries, 2014–2024 (n = 314)

There was a marked seasonality (Figure 4). Most cases were found in the late summer and early autumn months, corresponding well with the epidemiology of impetigo in general [5,6]. This pattern was seen in the Netherlands in 2019 and again from 2022 and onwards, and in Denmark the situation was the same in 2023 when the clone was first detected and again in 2024. It is therefore of importance to monitor the situation in the coming months, not only in the Netherlands and in Denmark but in all countries, as the clone has shown the potential to spread to at least 11 European countries and, in several countries, to persist for many years.

Date of isolation not available for three cases.

## Standard control, hygiene and treatment measures 

Recommendations for treatment vary between countries. In general, washing and topical treatment with chlorhexidine, fusidic acid or mupirocin is the first choice while in severe cases, oral systemic antibiotic treatments can be considered, including di- or flucloxacillin, clarithromycin, clindamycin and amoxicillin/clavulanic acid. Therefore, the resistance to both fusidic acid and meticillin, and partially to lincosamides and macrolides, is worrisome as topical and oral systemic antimicrobial treatment options for these infections are limited. Children with symptoms of impetigo should be kept home from daycare and school until lesions have healed. 

## Discussion

We report a clone of FAR-MRSA ST121 which causes impetigo and other skin and soft tissue infections and which in the last decade has spread to at least 11 European countries. 

Historically, the multilocus sequence typing (MLST) CC121 lineage of S. aureus has been implied in many outbreaks of impetigo [7-9]. The ability of the FAR-MRSA clone to cause impetigo is likely to be related to the presence of the toxin genes *eta, etb and edinC* [10-13]. Tourism, business travel and migration have been shown to facilitate the spread of MRSA clones [14,15]. Visiting school, family and friends was considered to be the cause of the spread of the Dutch outbreak in the period 2016 to 2018 [2]. Fusidic acid is a widely used antibiotic for skin and soft tissue infections and may provide a selection pressure in favour of the clone [14].

Differences in the presence and detections of the clone probably reflect differences in national MRSA surveillance systems, which vary greatly between the countries from which isolates in this study were included. Differences in healthcare seeking behaviour and diagnostic practices could also be contributing factors. It is likely that additional cases of infections with this clone have been treated without microbiological sampling or caused self-limiting mild disease that did not require treatment. The reported number of cases in this study is thus expected to be a minimum number. As the Netherlands and the Nordic countries have similar surveillance systems in place for MRSA, it seems fair to assume that the clone is more widespread in the Netherlands, where one of the first cases occurred and in Denmark, and less widespread in Sweden, Norway and Finland.

The study only included sequences from countries who had identified MRSA isolates with characteristics of the FAR-MRSA and replied to our initial EpiPulse message. Surveillance for MRSA in other countries not represented in this study may not include MRSA from skin and soft tissue infections. A limitation of this study is therefore that there may be many more FAR-MRSA present in Europe than described here.

## Conclusion

It is important to monitor the further dissemination and spread of the FAR-MRSA clone, both in countries where it is already found and in countries where the clone has not yet been detected, so that measures for controlling further spread can be implemented in a timely manner. Depending on local practices, increased microbiological sampling from impetigo lesions should be considered, as well as routine antimicrobial susceptibility testing of *S. aureus* impetigo isolates to detect meticillin and fusidic acid resistance.

## Data Availability

Whole-genome sequences from 94 Danish isolates were uploaded to The European Nucleotide Archive with accession numbers ERR ERR15284035–ERR15284128. Whole genome sequences from other countries were provided by participating institutions. When sequences have been uploaded to the National Center for Biotechnology Information, accession numbers are found in Supplementary Tables S1 and S3. Requests for the remaining sequences should be directed to the national reference laboratories.

## Supplementary Data

64000 Supplement
